# Application of Machine Learning to Predict Grain Boundary Embrittlement in Metals by Combining Bonding-Breaking and Atomic Size Effects

**DOI:** 10.3390/ma13010179

**Published:** 2020-01-01

**Authors:** Xuebang Wu, Yu-xuan Wang, Kan-ni He, Xiangyan Li, Wei Liu, Yange Zhang, Yichun Xu, Changsong Liu

**Affiliations:** 1 Key Laboratory of Materials Physics, Institute of Solid State Physics, Chinese Academy of Sciences, Hefei 230031, China; wyx811@mail.ustc.edu.cn (Y.-x.W.); kangnh@mail.ustc.edu.cn (K.-n.H.); xiangyanli@issp.ac.cn (X.L.); wliu@issp.ac.cn (W.L.); yangezhang@issp.ac.cn (Y.Z.); xuyichun@issp.ac.cn (Y.X.); 2 Department of Materials Science and Engineering, University of Science and Technology of China, Hefei 230026, China

**Keywords:** grain boundary embrittlement, machine learning, strengthening energy, support vector machine, artificial neural network

## Abstract

The strengthening energy or embrittling potency of an alloying element is a fundamental energetics of the grain boundary (GB) embrittlement that control the mechanical properties of metallic materials. A data-driven machine learning approach has recently been used to develop prediction models to uncover the physical mechanisms and design novel materials with enhanced properties. In this work, to accurately predict and uncover the key features in determining the strengthening energies, three machine learning methods were used to model and predict strengthening energies of solutes in different metallic GBs. In addition, 142 strengthening energies from previous density functional theory calculations served as our dataset to train three machine learning models: support vector machine (SVM) with linear kernel, SVM with radial basis function (RBF) kernel, and artificial neural network (ANN). Considering both the bond-breaking effect and atomic size effect, the nonlinear kernel based SVR model was found to perform the best with a correlation of *r*^2^ ~ 0.889. The size effect feature shows a significant improvement to prediction performance with respect to using bond-breaking effect only. Moreover, the mean impact value analysis was conducted to quantitatively explore the relative significance of each input feature for improving the effective prediction.

## 1. Introduction

Segregation-induced changes in grain boundary (GB) cohesion are often the controlling factor limiting the mechanical properties of metallic alloys. A small amount of solute atoms may significantly alter fracture toughness and corrosion of metallic alloys by orders of magnitude [1,2,3]. To evaluate the strengthening or weakening effect of segregants on GB cohesion, one prevalent approach is to calculate the so-called strengthening energy or embrittling potency, Δ*E_SE_*, of a particular segregated impurity, which is the segregation energy difference between a GB and a fracture free surface (FS) using the Rice-Wang model [4,5]. The value of Δ*E_SE_* plays a key role in the GB embrittlement or strengthening because there is a positive correlation between Δ*E_SE_* and the experimental shift of ductile-to-brittle transition temperature (DBTT) that could be used for the design of new alloys [6]. During the last few decades, based on accurate first-principle calculations, an intensive effort has been focused on quantification of the segregation-induced changes of GB cohesion and a large amount of quantitative computational data have been accumulated in different materials, such as Fe [7,8,9,10,11,12,13], Al [5,14,15], Ni [1,8,16,17,18], W [19], and Mo [20,21,22].

On the other hand, a few phenomenological models have been developed to understand and predict the solute-induced changes in GB cohesion. An earlier simple bond-breaking model was proposed by Seah to describe Δ*E_SE_* of different solutes in Fe GBs [23]. Geng et al. related Δ*E_SE_* in Fe and Ni GBs via a modified bond-breaking model with an added elastic mismatch term, and proved the predictions with rigorous first-principles results [8]. A recent study by Gibson and Schuh reviewed the existing data of 400 calculations and found that the simple bond-breaking model is robust to describe the solute-induced changes in GB cohesion [24]. The values of Δ*E_SE_* show a strong and positive correlation with the difference in cohesive energies (Δ*C*), while the contribution to embrittlement from other factors such as atomic size effects and charge transfer are secondary [24]. In addition, Lejcek et al. also reviewed the interfacial segregation and GB embrittlement in Ni and Fe and found that Δ*E_SE_* could be determined by the difference of sublimation energies of the solute and solvent (Δ*H*), which is in accordance with the analytical work of Seah [25,26]. The strengthening energy generally increases with increasing Δ*H* albeit in individual cases some exceptions can be found [25,26]. Very recently, Gibson and Schuh developed a quantitative model for Δ*E_SE_* under conditions of equilibrium segregation and proposed a GB cohesion map to predict whether a given solute–solvent pair will exhibit weakening or strengthening of GBs [27]. Except the similar common feature of Δ*C* or related quantities with previous modes, the ratio of bonding energies between the solute and solvent, captured by the ratio of their surface energies (RS), was emphasized in their model [27]. Instead of the traditional one-factor bond-breaking model that relates Δ*E_SE_* with relative cohesive energy, Tran et al. used a simple two-factor linear model described by the relative metallic radii and the relative difference in cohesive energy, and found it is able to account for most of the variations in the Δ*E_SE_* with a value of *r*^2^ > 0.79 [28].

The above semi-empirical models and accurate first-principles calculations significantly advance the understanding of solute-induce changes of GB cohesion undoubtedly. However, these methods are limited either in terms of their accuracy or high computational cost. Furthermore, the identification of key features in determining Δ*E_SE_* is far from trivial. Thus, the underlying mechanism in Δ*E_SE_* of solutes in different metallic GBs is not well understood. As there exists a quite extended amount of the accurate values of Δ*E_SE_* in the literature from density functional theory (DFT) calculations, it could enable us to make such a broad, quantitative analysis and prediction of Δ*E_SE_* using machine learning methods. The machine learning is one kind of statistical analysis method to capture the complex internal relationships by learning from empirical data [29] and gradually becomes a significant tool in the physics and material research [30]. Some common methods of machine learning include Support Vector Machine (SVM) [31,32], Artificial Neural Network (ANN) [33,34], Random Forest [35], and so on. The machine learning has been widely used to predict fundamental properties such as the formation enthalpy [36], solid solubility [37], solute diffusion [38], and lattice thermal conductivity [39] from DFT calculations and experimental vales [40]. Recently, the structures and energies of clean GBs in Cu and Al by using machine learning methods [41,42,43]. Zhu et al. reveal new ground states and multiple GBs in Cu by unsupervised machine learning post-processing analysis [41]. Gomberg et al. applied machine learning to connect the GB macro degrees of freedom and energy in asymmetric tilt GBs in Al and the models show a good prediction for GB energies [42]. Tamura et al. proposed a new scheme based on machine learning to predict atomic energies and GB energies in Al symmetric tilt GBs [43]. However, to date, no report has addressed the prediction of GB embrittlement in metals by solute segregation using machine learning methods.

In this work, we apply machine learning to predict Δ*E_SE_* of solutes in different metallic GBs including Ni, Fe, Al, W, Mo, W, and Zr, using easily available atomic and elemental properties of the constituting atoms, known as features or descriptors. Three machine learning algorithms are considered, including support vector machine (SVM) with linear kernel, SVM with radial basis function (RBF) kernel, and artificial neural network (ANN). We use standard statistical analysis methods to determine the factors and predict Δ*E_SE_* of solutes in different host metals. Our purpose is to develop fast and accurate models for prediction of Δ*E_SE_* and uncover the key features.

## 2. Methods

**Figure 1:**
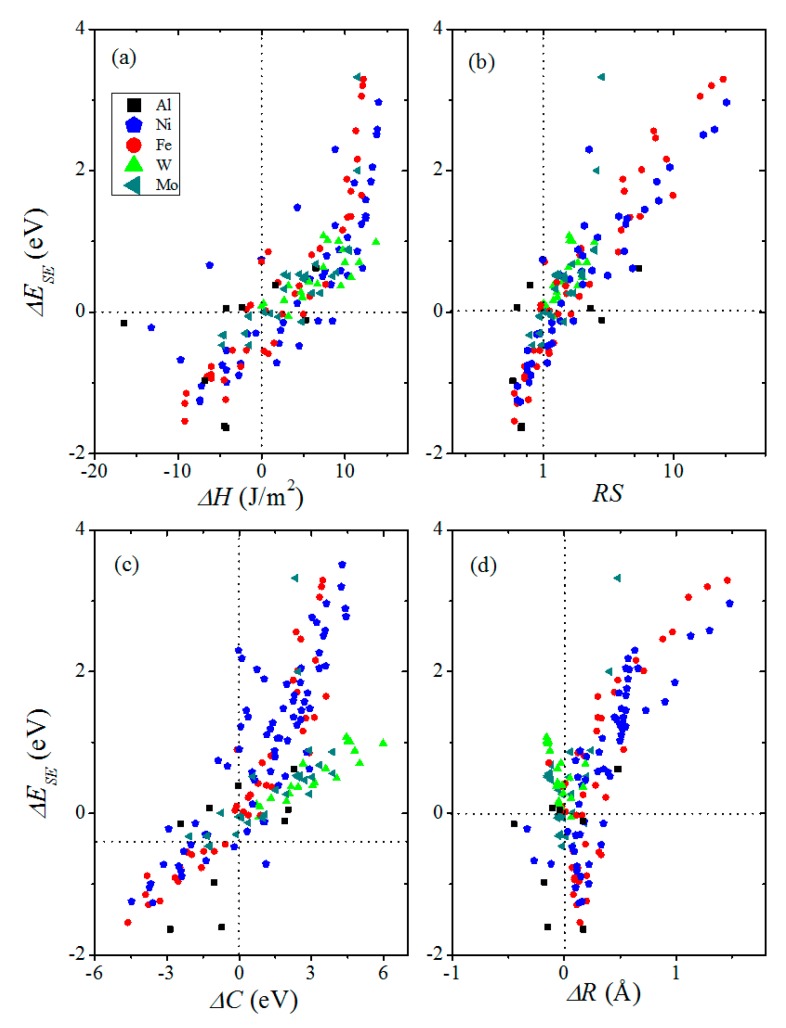
Trend of strengthening energies Δ*E_SE_* plotted against (**a**) difference of sublimation enthalpies Δ*H*, (**b**) ratio of the surface energies RS, (**c**) difference of cohesive energies Δ*C*, and (**d**) difference of atomic radii Δ*R* between the host and the segregated solute atoms. Data are from the aggregated data set.

The dataset we use for training and testing contains 142 data points by DFT calculations collected from literature [8,19,25] and first principles calculations, see Supplementary Table S1. The set contains 5 hosts which are Ni, Fe, W, Al, and Mo. To build accurate and reliable machine learning models, it is important to include relevant features that collectively capture the trends in the Δ*E_SE_* across the different metals. Based on the input parameters of the above models and by taking into account the accessibilities of the parameters, we select the difference of cohesive energies (Δ*C*) between the host and the segregated solute atoms, the ratio of their surface energies (RS), and the difference of sublimation enthalpies (Δ*H*) as chemical input parameters. For structural input parameters, the difference of atomic radii (ΔR) between the hosts and solutes is adopted. The values of chemical and structural features are provided in Table S1 of the Supplementary Material. Note that all the chemical and structural parameters can be obtained without performing any first principles calculations. A scatter-plot of the relationship between Δ*E_SE_* and Δ*C*, RS, Δ*H*, and Δ*R* is shown in Figure 1. It is clear from the figure that Δ*E_SE_* is positively correlated with Δ*C*, RS, and Δ*H*, indicating that the bond-breaking effect plays an important role in GB embrittlement. In addition, the atomic size effect also plays a non-negligible role because it is also well correlated with Δ*E_SE_*.

As discussed above, the correlation between bond-breaking effect and size effect in the context of GB embrittlement motivate the use of machine learning models to analyze the extent to which each of these phenomena is correlated with Δ*E_SE_*, once the other is taken into account. To asses this question, and to see if other variables are associated with Δ*E_SE_*, three machine learning models are constructed including SVM with linear kernel, SVM with RBF kernel, and ANN [31,44]. For SVM, there are many types of kernel function that can affect the performance of predictions and what we use in this work are two common ones, linear and radial basis function (RBF) [31,32]. To realize the SVM modeling, we consider the ε-SVR method using the LIBSVM package in MATLAB (version 9.3.0.713579, R2017b, MathWorks, Inc., Natick, MA, USA) [45]. For ANN, the type of network model we use in this work is the common one based on the back-propagation learning algorithm with Bayesian regularization [46,47], and operated via the toolbox called “nntool” in MATLAB.

The prediction performance of these methods is evaluated by four metrics, which are generally used as the error statistical parameters, mean absolute error (MAE), root mean square error (RMSE), standard deviation of error (SDE), and square correlation coefficient (*r*^2^). MAE is defined as

(1) $$MAE\;=\;\frac{1}{l}\;\sum_{l}^{i=1}\left|f\left(x_{i}\right)-y_{i}\right|.$$

RMSE is defined as

(2) $$RMSE=\sqrt{\frac{1}{l}\sum_{i=1}^{l}{\left(f\left(x_{i}\right)-y_{i}\right)}^{2}}.$$

SDE is defined as

(3) $$SDE=\;\sqrt{\frac{1}{l}\sum_{i=1}^{l}{\left(\left|f\left(x_{i}\right)-y_{i}\right|-MAE\right)}^{2}}.$$

Square correlation coefficient *r*^2^ is defined as

(4) $$r^{2}=\;{\left(\frac{\sum_{i=1}^{l}\left(f\left(x_{i}\right)-\bar{f\left(x\right)}\right)\left(y_{i}-\bar{y}\right)}{\sqrt{\sum_{i=1}^{l}{\left(f\left(x_{i}\right)-\bar{f\left(x\right)}\right)}^{2}}\sqrt{\sum_{i=1}^{l}{\left(y_{i}-\bar{y}\right)}^{2}}}\right)}^{2},$$

where l is data size, $y_{i}$ is the value of DFT calculations, and $f\left(x_{i}\right)$ is the predicted value of corresponding input. $\bar{f\left(x\right)}=\;\frac{1}{l}\;\sum_{i=1}^{l}f\left(x_{i}\right)$ (analogously for $\bar{y}$) and r is Pearson correlation coefficient [48].

## 3. Results and Discussion

**Figure 2:**
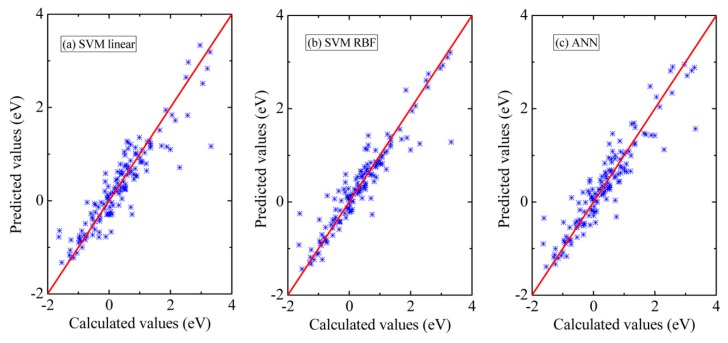
Comparison of Δ*E_SE_* from the density functional theory (DFT) calculations and the full-fit results from the three machine learning models with four input features. (**a**) Support vector machine (SVM) model with linear kernel, (**b**) SVM model with radial basis function (RBF) kernel, and (**c**) artificial neural network (ANN).

Figure 2 shows the fitted results from each of three machine learning methods compared against the DFT calculations using the above four features. These results are from using the entire dataset as the training data for each machine learning method. For the prediction using SVM with linear kernel in Figure 2a, a grid optimization method is used to optimize C, and the values of C range from 2^−10^ to 2^10^ and the step size is 0.5. The SVM model of the best prediction performance was found when C = 2^3.5^. The linear regression results are given by Δ*E_SE_* = −0.238980475 + 0.024468378 Δ*H* + 0.033997136 RS + 0.223070248 Δ*C* +1.05643494 Δ*R*. For the prediction using SVM with RBF kernel in Figure 2b, two parameters, *C* and *γ*, can be changed to optimize the model. Similarly, the grid optimization method is adopted to optimize *C* and *γ* ranging from 2^−10^ to 2^10^, respectively. The best parameters are found when *C* = 2^4^ and *γ* = 2^0.5^. For the prediction using ANN in Figure 2c, we use one input layer, one hidden layer including 4 hidden units and the output layer through the pre-test. The results of RMSE and *r*^2^ for three models are summarized in Table 1. It can be seen that the SVM model with RBF kernel and the ANN model show better performances than the SVM model with a linear kernel.

**Figure 3:**
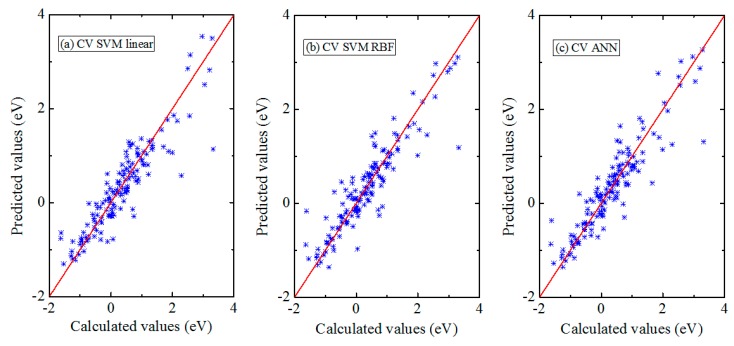
Comparison of Δ*E_SE_* from the DFT calculations and the 10-fold cross validation prediction results using (**a**) SVM model with linear kernel, (**b**) SVM model with RBF kernel, and (**c**) ANN.

Note that a good prediction for the training dataset does not suggest that the model has good prediction ability for the unknown dataset. Thus, to increase reliability of method, the data is randomly divided into 10 groups and during 10 iterations one group is set as the testing dataset, while the remaining nine groups are set as the training dataset. This process is called the 10-fold cross validation that can eliminate the chance of over-fitting [48,49]. For the SVM models with linear kernel and RBF kernel, we follow the similar optimization of model parameters for every group predictions. The values of parameters *C* and *γ* are listed in Table 2. For the stochastic nature of ANN model, the prediction procedure of every group is repeated 30 times and an average of 30 times predictions is taken. The prediction results of three models with all four features are shown in Figure 3. A comparison of MAE, RMSE, SDE, and *r*^2^ from the three models is also displayed in Table 1. Generally, the error of cross-validation procedure should always be higher than the error of fitted procedure and this is this case here. It can be seen from Table 1 that, for all three models, the values of MAE, RMSE, SDE, and *r*^2^ are in the range of 0.280–0.300 eV, 0.409–0.424 eV, 0.290–0.300 eV, and 0.827–0.839, respectively. This indicates that the cross-validation errors from three methods using the four features are comparable. Moreover, to increase the accuracy of the models, the 14-fold cross validation tests are also performed, and the prediction results are shown in the Supplementary Material. Generally, the prediction results are similar to that using 10-fold cross validation, suggesting that the latter is sufficient to ensure accuracy.

**Figure 4:**
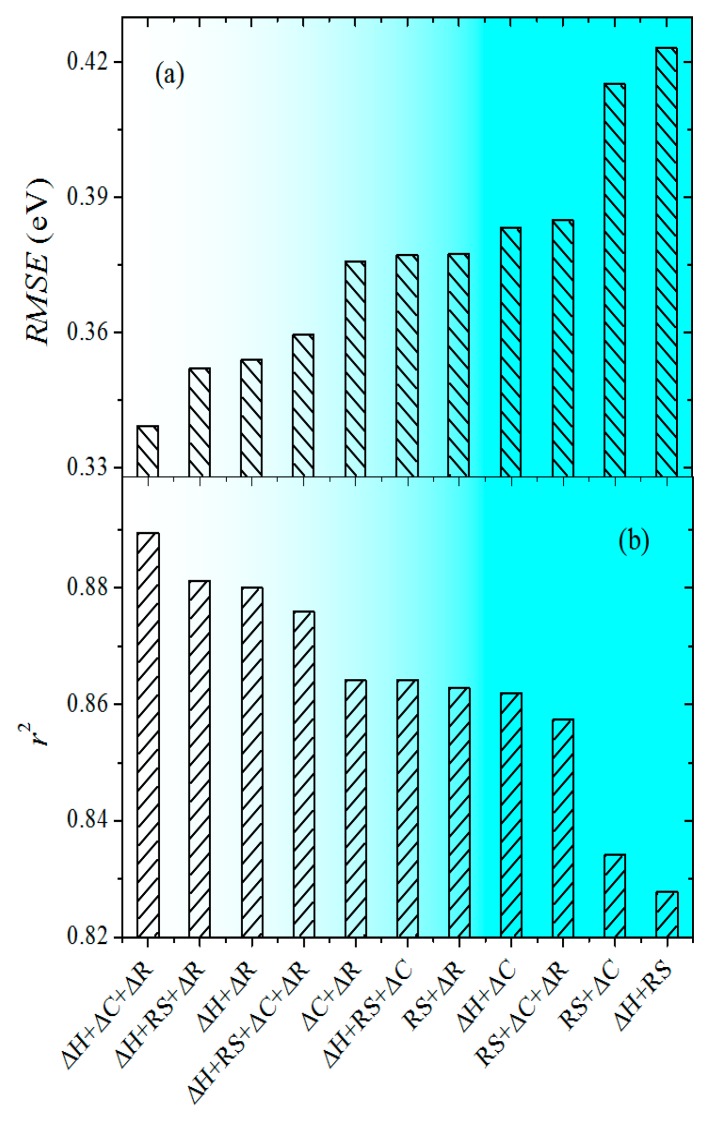
Comparison of values of (**a**) root mean square error (RMSE) and (**b**) *r*^2^ of the SVM models with RBF kernel for different input features.

To analyze the effect of features on the Δ*E_SE_*, the SVM models with RBF kernel are used with different combinations of input features. For each set of features, the SVM models are optimized with full fitting. The values of RMSE and *r*^2^ of the SVM models with different input features are displayed in Figure 4. The nonlinear SVM model including Δ*H*, Δ*C*, and Δ*R* is found to be the best description for Δ*E_SE_*, with RMSE of 0.339 eV and *r*^2^ of 0.889, while the worst performance is obtained using Δ*H* and RS, with RMSE of 0.423 eV and *r*^2^ of 0.828, respectively. The correlation among Δ*H*, RS, and Δ*C* can be inferred from the fact that the values of RMSE and *r*^2^ do not add to one another when the regressors are combined in the same model. However, the bond breaking effects reflected by Δ*H*, RS, and Δ*C* are clearly demonstrated as significant in GB cohesion. It should be noted that, when removing the feature Δ*R* from the groups of Δ*H* + RS + Δ*C* + Δ*R*, Δ*H* + Δ*C* + Δ*R*, Δ*H* + RS + Δ*R*, and RS + Δ*C* + Δ*R*, the performance becomes poorer in different degrees. The analysis shows that Δ*R* is a significant feature to the contribution of Δ*E_SE_*. Therefore, the statistical conclusion shows that bond-breaking and atomic size effects are independent and substantial contributors to GB cohesion.

**Figure 5:**
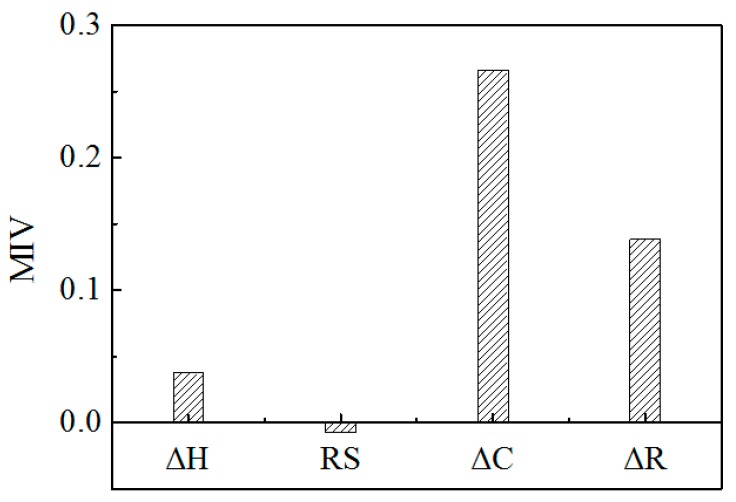
The mean impact values (MIV) for input features Δ*H*, RS, Δ*C*, and Δ*R*.

To quantitatively explore the relative significance of each input variable for improving the prediction performance, the mean impact value (MIV) analysis was conducted using the similar method previously by Jiang et al. [50] and Liu et al. [51]. The MIV values for each input variable on each output variable are calculated and shown in Figure 5. It can be found that the important sequence of the factors for the strengthening energies is △*C* > △*R* > △*H* > RS. △*C* and △*R* are the two most important factors to influence the strengthening energies. This may explain why the two-factor model is able to account for most of the variation in the strengthening energies in Mo GBs [28]. As shown in Figure 5, the features △*H*, △*C* and △*R* show a positive correlation with grain boundary embrittlement, while RS appears a negative correlation. According to the MIV analysis, it can be found that △*H*, △*C*, and △*R* have a high correlation with grain boundary embrittlement. This is consistent with the above conclusion that the SVM model with these three features yields the best results amongst all the test models.

The aggregation of Δ*E_SE_* in different host metals from so many sources should be expected to lead to significant scatter due to the different computations such as potential selection and relaxation standard [52]. Here, the relative good performance of nonlinear SVM model using Δ*H*, Δ*C*, and Δ*R* is thus interpreted to be highly physically meaningful. The present model could not only be used to understand GB strengthening or embrittlement and its underlying physical origins, but also serves as a quantitative prediction of the strengthening energies by solute segregation in other systems that have not yet been studied experimentally or computationally, which is helpful for the rational design and screening of novel materials with desired mechanical properties for specific applications.

## 4. Conclusions

In this work, we have trained three separate machine learning models to infer the driving forces for GB embrittlement and predict strengthening energies for impurities in different host metals. The readily accessible features (i.e., difference of sublimation enthalpies ΔH, ratio of surface energies RS, difference of cohesive energies Δ*C* and difference of atomic radii Δ*R*) are chosen as descriptors. It was shown that the energetics of embrittlement is quantitatively described by two simple effects: bond-breaking and atomic size. A nonlinear kernel based support vector regression model with features Δ*H*, Δ*C*, and Δ*R* shows the best performance prediction for the aggregated set of strengthening energies, with RMSE of 0.339 eV and *r*^2^ of 0.889. The feature of the size effect was found to exhibit considerable importance to the model prediction. Additionally, the MIV based analysis has been carried out for evaluating the features’ importance. Results show that the important sequence of the factors for the GB embrittlement is: Δ*C* > Δ*R* > Δ*H* > RS. The methods employed in the present work, i.e., clarifying the physical mechanism and extracting the key feature quantities as descriptors by first principles calculations and then predicting material properties via machine learning, can be extended to predict other material properties.

## Figures and Tables

**Table 1:** Values of mean absolute error (MAE), root mean square error (RMSE), square correlation coefficient (SDE) and *r*^2^ from full fit and 10-fold cross validation predictions of three machine learning models with four input features. SVM: support vector machine; RBF: radial basis function; ANN: artificial neural network.

Methods	Metrics	SVM with Linear Kernel	SVM with RBF Kernel	ANN
Full fitting	MAE (eV)	0.286	0.233	0.265
	RMSE (eV)	0.406	0.359	0.367
	SDE (eV)	0.288	0.274	0.254
	*r* ^2^	0.843	0.876	0.870
10-fold CV	MAE (eV)	0.300	0.280	0.288
	RMSE (eV)	0.424	0.414	0.409
	SDE (eV)	0.300	0.305	0.290
	*r* ^2^	0.827	0.835	0.839

**Table 2:** The values of parameter *C* for SVM model with linear kernel and parameters *C* and *γ* for SVM model with an RBF kernel.

Group	SVM with Linear Kernel	SVM with RBF Kernel	
	*C*	*C*	*γ*
G1	11.3137085	11.3137085	22.627417
G2	11.3137085	16	2
G3	8	8	2
G4	64	16	11.3137085
G5	11.3137085	2	4
G6	32	11.3137085	2
G7	362.038672	2.82842712	4
G8	16	11.3137085	2
G9	11.3137085	11.3137085	11.3137085
G10	4	32	8

## Supplementary Materials

The data related to this article are available in the https://www.mdpi.com/1996-1944/13/1/179/s1. Figure S1: Comparison of Δ*E_SE_* from DFT calculations and 14-fold cross validation prediction results using three machine learning models; Table S1: Statistical parameters from full fit and 14-fold cross validation predictions of three machine learning models; Table S2: Database on Δ*E_SE_* of solutes in different host metals for the training and test of the machine learning models; Table S3: Statistical parameters for the SVM model with RBF kernel with different features; Table S4: Statistical parameters for three machine learning models with best performance using different features.
