# Supplementary material for: Application of Machine Learning to Predict Grain Boundary Embrittlement in Metals by Combining Bonding-Breaking and Atomic Size Effects
Source: Materials (Basel). 2020 Jan 1;13(1):179. doi: 10.3390/ma13010179 (PMC6981756; doi:10.3390/ma13010179)
Supplement: Supplementary file 1 [file materials-13-00179-s001.pdf]

materials

Article

Application of Machine Learning to Predict Grain Boundary Embrittlement in Metals by Combining Bonding-Breaking and Atomic Size Effects

Xuebang Wu [1,*], Yu-xuan Wang [1,2], Kan-ni He [1,2], Xiangyan Li [1], Wei Liu [1], Yange Zhang [1], Yichun Xu [1] and Changsong Liu [1,*]

1. Key Laboratory of Materials Physics, Institute of Solid State Physics, Chinese Academy of Sciences, Hefei 230031, China; wyx811@mail.ustc.edu.cn (Y.W.); kangnh@mail.ustc.edu.cn (K.H.); xiangyanli@issp.ac.cn (X.L.); wliu@issp.ac.cn (W.L.); yangezhang@issp.ac.cn (Y.Z.); xuyichun@issp.ac.cn (Y.X.)
2. Department of Materials Science and Engineering, University of Science and Technology of China, Hefei 230026, China

* Correspondence: xbwu@issp.ac.cn (X.W.); csliu@issp.ac.cn (C.L.)

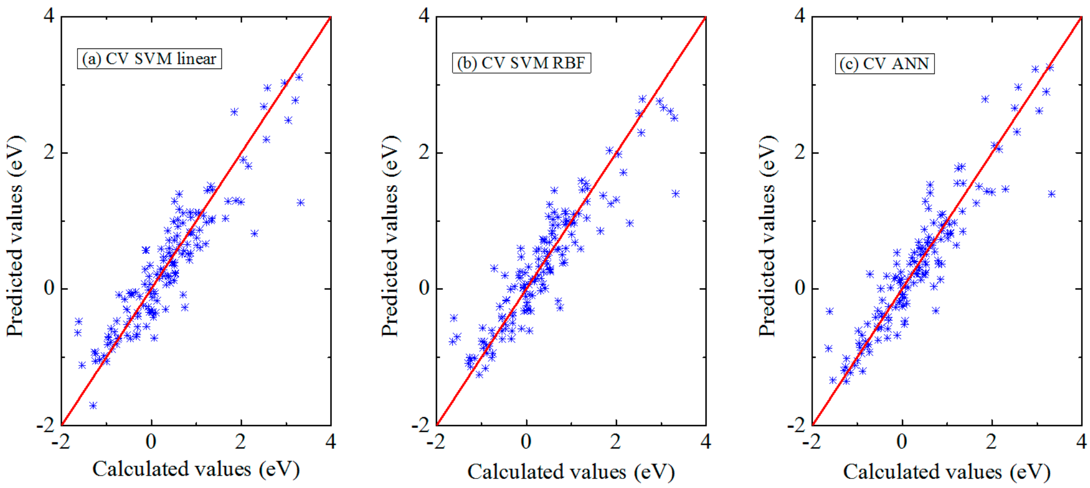

Figure S1. Comparison of ΔE_{SE} from the DFT calculations and the 14-fold cross validation prediction results using (**a**) SVM model with linear kernel; (**b**) SVM model with RBF kernel, and (**c**) ANN.

Table S1. Values of MAE, RMSE, SDE and r^2 from full fit and 14-fold cross validation predictions of three machine learning models with four input features.

-	-	SVM with linear kernel	SVM with RBF kernel	ANN
Full fitting	MAE(eV)	0.286	0.233	0.265
	RMSE (eV)	0.406	0.359	0.367
	SDE(eV)	0.288	0.274	0.254
	r^2	0.843	0.876	0.870
14-fold CV	MAE(eV)	0.310	0.304	0.278
	RMSE (eV)	0.419	0.415	0.390
	SDE(eV)	0.282	0.283	0.274
	r^2	0.831	0.835	0.844

Table S2. Database on strengthening energies (ΔE_{SE}) of various solutes in different host metals for the training and test of the machine learning models. ΔH is the difference of sublimation enthalpies between the host and the segregated solute atoms, RS is the ratio of surface energies, ΔC is the difference of cohesive energies, and ΔR is the difference of atomic radii.

-	Host	Solute	ΔH (Jm^{-2})	RS	Δ*C* (eV)	Δ*R* (Å)	ΔE_{SE} (eV)	Ref.
1	Ni	Cs	14	25.16428	3.636	1.48	2.96418	[1]
2	Ni	Rb	13.9	20.49535	3.588	1.3	2.5807	[1]
3	Ni	K	13.8	16.78938	3.506	1.13	2.50815	[1]
4	Ni	Na	13.3	9.2416	3.327	0.66	2.05213	[1]
5	Ni	Ba	13.1	7.37698	2.54	0.99	1.84484	[1]
6	Ni	Tl	12.5	4.40093	2.56	0.47	1.32663	[1]
7	Ni	Bi	12.5	3.76966	2.26	0.45	1.35772	[1]
8	Ni	Mg	12.1	4.8361	2.93	0.35	0.62186	[1]
9	Ni	Pb	12	4.28	2.41	0.5	1.24371	[1]
10	Ni	Li	11.5	4.16514	2.81	0.31	0.86023	[1]
11	Ni	Sb	10.3	2.6132	1.69	0.34	1.05716	[1]
12	Ni	In	10.3	3.10623	1.92	0.41	0.51821	[1]
13	Ni	Sn	9.5	2.37253	1.3	0.3	0.59076	[1]
14	Ni	As	9.3	1.85503	1.48	0.14	0.88096	[1]
15	Ni	Y	8.8	2.05661	0.07	0.55	1.22298	[1]
16	Ni	La	8.8	2.24627	−0.03	0.63	2.30087	[1]
17	Ni	Ga	8.3	2.00952	1.63	0.16	0.39384	[1]
18	Ni	Ag	7.8	2	1.49	0.2	0.79805	[1]
19	Ni	Sc	7.6	1.98337	0.54	0.39	0.5804	[1]
20	Ni	Ge	7.3	1.38204	0.59	0.12	0.50785	[1]
21	Ni	Al	6.8	1.71296	1.05	0.18	−0.12437	[1]
22	Ni	Au	5.8	1.58665	0.63	0.19	0.46639	[1]
23	Ni	Si	4.5	1.07013	−0.19	0.07	−0.47676	[1]
24	Ni	Pd	4.3	1.36734	0.55	0.13	0.12437	[1]
25	Ni	Zr	2.6	1.16291	−1.81	0.35	−0.1451	[1]
26	Ni	Cr	2.3	1.17196	0.34	0.03	−0.25911	[1]
27	Ni	Hf	2.1	1.15761	−2	0.33	−0.44566	[1]
28	Ni	Be	1.8	1.07377	1.12	−0.12	−0.71513	[1]
29	Ni	V	0	0.98954	−0.87	0.1	0.74623	[1]
30	Ni	Pt	−0.7	0.93837	−1.4	0.14	−0.30056	[1]
31	Ni	Rh	−1.7	0.88782	−1.31	0.1	−0.31093	[1]
32	Ni	Nb	−2.5	0.81084	−3.13	0.22	−0.7255	[1]
33	Ni	Mo	−2.7	0.80432	−2.38	0.15	−0.89133	[1]
34	Ni	Tc	−4.2	0.75525	−2.41	0.11	−0.81878	[1]
35	Ni	Ru	−4.2	0.75669	−2.3	0.09	−0.53894	[1]
36	Ni	Ta	−4.2	0.77766	−3.66	0.22	−0.99497	[1]
37	Ni	Ir	−4.7	0.75618	−2.5	0.11	−0.74623	[1]
38	Ni	Os	−7.2	0.63353	−3.73	0.1	−1.04679	[1]
39	Ni	W	−7.4	0.63397	−4.46	0.16	−1.24371	[1]
40	Ni	Re	−7.4	0.66338	−3.59	0.13	−1.26444	[1]
41	Fe	Li	9.7	3.95563	2.65	0.29	1.16	[1]
42	Fe	Be	0	1.01975	0.96	−0.14	0.71	[1]
43	Fe	Na	11.5	8.77672	3.17	0.64	2.16	[1]
44	Fe	Mg	10.3	4.59284	2.77	0.33	1.34	[1]
45	Fe	Al	5	1.6268	0.89	0.16	−0.03	[1]
46	Fe	K	12	15.94483	3.346	1.11	3.05	[1]
47	Fe	Sc	5.8	1.8836	0.38	0.37	0.22	[1]
48	Fe	Ti	1.5	1.20115	−0.57	0.19	−0.44	[1]
49	Fe	V	−1.8	0.93976	−1.03	0.08	−0.54	[1]
50	Fe	Cr	0.5	1.11301	0.18	0.01	0.02	[1]
51	Fe	Mn	4.5	1.48788	1.36	−0.01	0.37	[1]
52	Fe	Co	−1.3	0.95866	−0.11	−0.02	0.1	[1]

53	Fe	Ni	−1.8	0.9497	−0.16	−0.02	0.04	[1]
54	Fe	Cu	2	1.2685	0.79	0.01	0.42	[1]
55	Fe	Zn	0.8	3.75584	2.93	0.12	0.85	[1]
56	Fe	Rb	12.1	19.46438	3.428	1.28	3.2	[1]
57	Fe	Y	7	1.95315	−0.09	0.53	0.9	[1]
58	Fe	Zr	0.8	1.10441	−1.97	0.33	−0.59	[1]
59	Fe	Nb	−4.3	0.77005	−3.29	0.2	−1.24	[1]
60	Fe	Mo	−4.5	0.76386	−2.54	0.13	−0.96	[1]
61	Fe	Tc	−6	0.71726	−2.57	0.09	−0.94	[1]
62	Fe	Ru	−6	0.71863	−2.46	0.07	−0.77	[1]
63	Fe	Rh	−3.5	0.84316	−1.47	0.08	−0.54	[1]
64	Fe	Pd	2.5	1.29856	0.39	0.11	−0.03	[1]
65	Fe	Ag	6	1.8994	1.33	0.18	0.81	[1]
66	Fe	Cd	10.7	5.50849	3.12	0.3	1.35	[1]
67	Fe	Cs	12.2	23.89845	3.476	1.46	3.29	[1]
68	Fe	Ba	11.3	7.0059	2.38	0.97	2.56	[1]
69	Fe	Hf	0.3	1.09938	−2.16	0.31	−0.55	[1]
70	Fe	Ta	−6	0.73854	−3.82	0.2	−0.88	[1]
71	Fe	W	−9.2	0.60208	−4.62	0.14	−1.54	[1]
72	Fe	Re	−9.2	0.63001	−3.75	0.11	−1.29	[1]
73	Fe	Os	−9	0.60166	−3.89	0.08	−1.15	[1]
74	Fe	Ir	−6.5	0.71814	−2.66	0.09	−0.91	[1]
75	Fe	Pt	−2.5	0.89117	−1.56	0.12	−0.77	[1]
76	Fe	Au	4	1.50684	0.47	0.17	0.26	[1]
77	Fe	Hg	12	9.86495	3.61	0.3	1.65	[1]
78	Fe	Tl	10.7	4.17955	2.4	0.45	1.71	[1]
79	Fe	Pb	10.2	4.0647	2.25	0.48	1.88	[1]
80	Fe	Sn	7.7	2.25318	1.14	0.28	0.39384	[1]
81	W	Ti	10.7	1.995	4.05	0.05	0.49368	[2]
82	W	V	7.4	1.56086	3.59	−0.06	0.63093	[2]
83	W	Cr	9.7	1.84861	4.8	−0.13	0.88263	[2]
84	W	Mn	13.7	2.47123	5.98	−0.15	0.98864	[2]
85	W	Fe	9.2	1.66091	4.62	−0.14	1.00787	[2]
86	W	Co	7.9	1.59224	4.51	−0.16	1.01817	[2]
87	W	Ni	7.4	1.57736	4.46	−0.16	1.07781	[2]
88	W	Zr	10	1.83433	2.65	0.19	0.70068	[2]
89	W	Nb	4.9	1.27899	1.33	0.06	0.21044	[2]
90	W	Mo	4.7	1.2687	2.08	−0.01	0.28081	[2]
91	W	Ru	3.2	1.19358	2.16	−0.07	0.37601	[2]
92	W	Rh	5.7	1.40041	3.15	−0.06	0.43334	[2]
93	W	Pd	11.7	2.15679	5.01	−0.03	0.70752	[2]
94	W	Hf	9.5	1.82597	2.46	0.17	0.37037	[2]
95	W	Ta	3.2	1.22665	0.8	0.06	−0.05256	[2]
96	W	Re	0	1.04639	0.87	−0.03	0.08718	[2]
97	W	Os	0.2	0.9993	0.73	−0.06	0.12823	[2]
98	W	Ir	2.7	1.19277	1.96	−0.05	0.16962	[2]
99	W	Pt	6.7	1.48015	3.06	−0.02	0.40042	[2]
100	W	Sb	7.7	2.24712	3.5	−0.01	1.2064	[2]
101	W	Li	8.9	3.58165	4.62	−0.04	1.06959	[2]
102	W	Sn	6.9	2.04016	3.11	−0.05	0.72343	[2]
103	W	Cd	9.9	4.98771	5.09	−0.03	0.58351	[2]
104	W	Si	1.9	0.92022	1.62	−0.28	0.31507	[2]
105	W	Ni	−2.6	0.85991	1.81	−0.35	0.05078	[2]
106	W	Hf	−0.5	0.99544	−0.19	−0.02	−0.00311	[2]
107	W	Nb	−5.1	0.69725	−1.32	−0.13	−0.10572	[2]
108	W	Cr	−0.3	1.00778	2.15	−0.32	−0.17101	[2]
109	W	Fe	−0.8	0.90546	1.97	−0.33	−0.34306	[2]
110	Al	Cr	−4.5	0.68417	−0.71	−0.15	−1.61268	[3]

111	Al	Mg	5.3	2.82324	1.88	0.17	−0.11401	[3]
112	Al	Na	6.5	5.3951	2.28	0.48	0.62186	[3]
113	Al	Ni	−6.8	0.58378	−1.05	−0.18	−0.97424	[3]
114	Al	Zn	−4.2	2.30873	2.04	−0.04	0.04975	[3]
115	Al	Zr	−4.2	0.67889	−2.86	0.17	−1.63755	[3]
116	Al	Si	−2.3	0.62472	−1.24	−0.11	0.06944	[3]
117	Mo	Sc	10.3	2.4659	2.92	0.24	0.88575	this work
118	Mo	Ti	6	1.57248	1.97	0.06	0.27243	this work
119	Mo	V	2.7	1.23028	1.51	−0.05	0.32965	this work
120	Mo	Cr	5	1.45709	2.72	−0.12	0.46928	this work
121	Mo	Mn	9	1.94785	3.9	−0.14	0.56104	this work
122	Mo	Fe	4.5	1.30914	2.54	−0.13	0.53624	this work
123	Mo	Co	3.2	1.25502	2.43	−0.15	0.51168	this work
124	Mo	Ni	2.7	1.24329	2.38	−0.15	0.53263	this work
125	Mo	Cu	6.5	1.66065	3.33	−0.12	0.68231	this work
126	Mo	Y	11.5	2.55696	2.45	0.4	1.99938	this work
127	Mo	Zr	5.3	1.44584	0.57	0.2	0.52031	this work
128	Mo	Nb	0.2	1.00811	−0.75	0.07	0.00363	this work
129	Mo	Tc	−1.5	0.93899	−0.03	−0.04	−0.05051	this work
130	Mo	Ru	−1.5	0.94079	0.08	−0.06	−0.06776	this work
131	Mo	Rh	1	1.10381	1.07	−0.05	−0.01622	this work
132	Mo	Pd	7	1.7	2.93	−0.02	0.2708	this work
133	Mo	Ag	10.5	2.48658	3.87	0.05	0.86421	this work
134	Mo	La	11.5	2.79277	2.35	0.48	3.3256	this work
135	Mo	Hf	4.8	1.43924	0.38	0.18	−0.13444	this work
136	Mo	Ta	−1.5	0.96686	−1.28	0.07	−0.46733	this work
137	Mo	W	−4.7	0.78821	−2.08	0.01	−0.32918	this work
138	Mo	Re	−4.7	0.82478	−1.21	−0.02	−0.46524	this work
139	Mo	Os	−4.5	0.78766	−1.35	−0.05	−0.31954	this work
140	Mo	Ir	−2	0.94015	−0.12	−0.04	−0.29895	this work
141	Mo	Pt	2	1.16667	0.98	−0.01	−0.06599	this work
142	Mo	Au	8.5	1.97267	3.01	0.04	0.51137	this work

Table S3. Values of RMSE and r^2 for the SVM model with RBF kernel with different combinations of input features.

**Amount**	**Feature**	**RMSE**	r^2
2	$\Delta H + \Delta R$	0.354	0.880
	$\Delta C + \Delta R$	0.375	0.864
	RS + ΔR	0.377	0.863
	$\Delta H + \Delta C$	0.383	0.862
	RS + ΔC	0.415	0.834
	ΔH + RS	0.423	0.828
3	$\Delta H + \Delta C + \Delta R$	0.339	0.889
	ΔH + RS + ΔR	0.352	0.881
	ΔH + RS + ΔC	0.377	0.864
	RS + $\Delta C + \Delta R$	0.385	0.857
4	ΔH + RS + $\Delta C + \Delta R$	0.359	0.876

Table S4. Values of RMSE and r^2 for three ML models with best performance using different combinations of features on strengthening energies.

Amount	Method	Features	RMSE	r^2
	L-SVR	$\Delta C + \Delta R$	0.398	0.848
2	RBF-SVR	$\Delta H + \Delta R$	0.354	0.880
	ANN	$\Delta H + \Delta R$	0.349	0.883
	L-SVR	$\Delta H + \Delta C + \Delta R$	0.397	0.849
3	RBF-SVR	$\Delta H + \Delta C + \Delta R$	0.339	0.889
	ANN	$\Delta H + \Delta C + \Delta R$	0.334	0.881
	L-SVR	$\Delta H + \Delta S + \Delta C + \Delta R$	0.406	0.843
4	RBF-SVR	$\Delta H + \Delta S + \Delta C + \Delta R$	0.359	0.876
	ANN	$\Delta H + \Delta S + \Delta C + \Delta R$	0.332	0.894

Reference

1. Geng, W.; Freeman, A. J.; Olson, G. B., Influence of alloying additions on grain boundary cohesion of transition metals: first-principles determination and its phenomenological extension. *Phys. Rev. B* **2001**, *63*, 165415.
2. Wu, X.; You, Y.-W.; Kong, X.-S.; Chen, J.-L.; Luo, G.-N.; Lu, G.-H.; Liu, C.; Wang, Z., First-principles determination of grain boundary strengthening in tungsten: dependence on grain boundary structure and metallic radius of solute. *Acta Mater.* **2016**, *120*, 315–326.
3. Gibson, M. A.; Schuh, C. A. A survey of ab-initio calculations shows that segregation-induced grain boundary embrittlement is predicted by bond-breaking arguments. *Scr. Mater.* **2016**, *113*, 55–58.

© 2020 by the authors. Licensee MDPI, Basel, Switzerland. This article is an open access article distributed under the terms and conditions of the Creative Commons Attribution (CC BY) license (http://creativecommons.org/licenses/by/4.0/).
